# Cardiovascular tissue regeneration system based on multiscale scaffolds comprising double-layered hydrogels and fibers

**DOI:** 10.1038/s41598-020-77187-8

**Published:** 2020-11-23

**Authors:** Yun-Min Kook, Soonjae Hwang, Hyerim Kim, Ki-Jong Rhee, Kangwon Lee, Won-Gun Koh

**Affiliations:** 1 Department of Chemical and Biomolecular Engineering, Yonsei University, 50 Yonsei-ro, Seodaemun-gu, Seoul, 120-749 Republic of Korea; 2 Program in Nanoscience and Technology, Graduate School of Convergence Science and Technology, Seoul National University, Seoul, Republic of Korea; 3 Advanced Institutes of Convergence Technology, Suwon, Gyeonggi-do Republic of Korea; 4 Department of Biomedical Laboratory Science, College of Health Sciences, Yonsei University at Wonju, Wonju, Gangwon-do 220-710 Republic of Korea; 5 Natural Product Informatics Research Center, Korea Institute of Science and Technology, Gangneung, Gangwon-do 25451 Republic of Korea

**Keywords:** Mesenchymal stem cells, Nanobiotechnology, Angiogenesis, Stem cells

## Abstract

We report a technique to reconstruct cardiovascular tissue using multiscale scaffolds incorporating polycaprolactone fibers with double-layered hydrogels comprising fibrin hydrogel surrounded by secondary alginate hydrogel. The scaffolds compartmentalized cells into the core region of cardiac tissue and the peripheral region of blood vessels to construct cardiovascular tissue, which was accomplished by a triple culture system of adipose-derived mesenchymal stem cells (ADSCs) with C2C12 myoblasts on polycaprolactone (PCL) fibers along with human umbilical vein endothelial cells (HUVECs) in fibrin hydrogel. The secondary alginate hydrogel prevented encapsulated cells from migrating outside scaffold and maintained the scaffold structure without distortion after subcutaneous implantation. According to in vitro studies, resultant scaffolds promoted new blood vessel formation as well as cardiomyogenic phenotype expression of ADSCs. Cardiac muscle-specific genes were expressed from stem cells and peripheral blood vessels from HUVECs were also successfully developed in subcutaneously implanted cell-laden multiscale scaffolds. Furthermore, the encapsulated stem cells modulated the immune response of scaffolds by secreting anti-inflammatory cytokines for successful tissue construction. Our study reveals that multiscale scaffolds can be promising for the remodeling and transplantation of cardiovascular tissue.

## Introduction

Despite the importance of cardiovascular tissue development and regeneration, successful methods that promote tissue construction have not been developed yet. Conventional surgical treatment for cardiovascular diseases such as cardiomyopathy using left ventricular assist device (LVAD) or heart transplantation often have some additional drawbacks after the operation because of limited durability in the long term^1–3^. Using tissue engineering approaches, the complexity of modeling cardiovascular tissue can be represented and controlled without critical risks to patients. Specifically, cell-based tissue regeneration therapies enable the fabrication of new functionalized cardiovascular tissue using various stem cells^4,5^. However, these cell-based approaches have some challenges for successful replacement of damaged cardiovascular tissues. First, large amounts of cells are required depending on the size of the defect to obtain clinically meaningful tissue regeneration, and in the case of hiPSC-derived cardiomyocytes, more than 10^9^ cells are required for clinically relevant regeneration^6–8^. Second, transplanted cells are required enhanced survival rate for long time with biomaterials (up to several weeks), electrical interaction with host tissue, and encouragement peripheral new blood vessel regeneration to maintain viability of transplanted cells^9,10^. Third, reduced host immune response is required for successful cell transplantation to the desired delivery site and interaction between delivered cells and host tissue^11–13^.

As one of the simplest cell transplantation trials, injection of cell suspension has been a good candidate to deliver cells into myocardial infarction (MI) sites. Although the direct intramyocardial injection of cells could recover small lesion area, however injected cells caused immune rejection, teratoma, and cancer^14,15^. Furthermore, several studies used hydrogel or hydrogel-based patch to regenerate MI site. However, without any cells or biomolecules, only hydrogel could not significantly upregulate several angiogenic and anti-inflammatory-related genes after transplantation to rat MI model^16^. Therefore, a new concept is required for successful cell transplantation systems capable of controlling a large number of stem cell differentiations, organizing peripheral blood vessel formation, and reducing immune response to enhance cardiovascular tissue regeneration. To satisfy these demands, the cell-based cardiovascular tissue repair trials using biomaterials or cell sheet have been reported recently. The efficacy of stem cell differentiation into cardiac lineages with polysaccharides patch and human iPSC-derived cardiac patch increased cardiovascular regeneration after transplantation into rat MI model^17,18^. Several studies have controlled stem cell differentiation into cardiac lineages in vitro and have improved MI by inducing angiogenesis after stem cell transplantation^19–21^ or enhanced cardiac tissue repair after myoblast sheet transplantation^3,17,22^. These studies have shown the potential of cell-based therapy for cardiac tissue repair; however, few studies were reported on the development of complete engineered whole cardiovascular tissue implants with peripheral blood vessels.

Herein, we developed multiscale scaffolds incorporating a triple culture system of adipose-derived mesenchymal stem cells (ADSCs) with C2C12 myoblasts on PCL fibers along with human umbilical vein endothelial cells (HUVECs) in fibrin hydrogel to develop in vitro engineered cardiovascular tissue as a newly designed tissue transplantation system. The resultant scaffolds consisting of PCL fibers and fibrin hydrogel were surrounded by alginate hydrogel layers to provide mechanical stability. We compartmentalized cells into the core region of cardiac tissue and the peripheral region of blood vessels to construct in vitro cardiovascular tissue, which enabled cardiovascular tissue formation in the multiscale scaffolds in vitro and after subcutaneous implantation. We also confirmed that the transplanted stem cells expressed cardiac muscle specific genes, reduced host immune responses by secreting immunosuppressive cytokines and modulated peripheral blood vessels. Our study thus provides a novel cell-based therapy and engineered tissue platform for cardiovascular tissue repair.

## Materials and methods

### Cell culture

Primary human adipose-derived mesenchymal stem cells (ADSCs) isolated from normal human adipose tissue (Approved by Ethics committee of CEFO, CB-IRB-120224) obtained by liposuction or surgical resection were purchased from CEFO (Seoul, Korea). Human umbilical vein endothelial cells (HUVECs) were purchased from Lonza Inc., and C2C12 myoblasts were purchased from the Korean Cell Line Bank (Seoul, Korea). Cells were cultured in T-75 flasks at 37 °C in a humid atmosphere containing 5% CO_2_ until used for experiments. Dulbecco’s modified Eagle’s minimum essential medium (DMEM) and minimum essential medium Eagle-alpha modification (Alpha MEM) with 10% fetal bovine serum and 1% antibiotic-antimycotics were to culture ADSCs and C2C12 cells, respectively. Endothelial basal medium (EBM-2, Lonza) with endothelial growth supplement Single Quots (EGM-2, Lonza) and 1% v/v antibiotic-antimycotics were used for the culture of HUVECs.

### Scaffold fabrication

**Figure 1:**
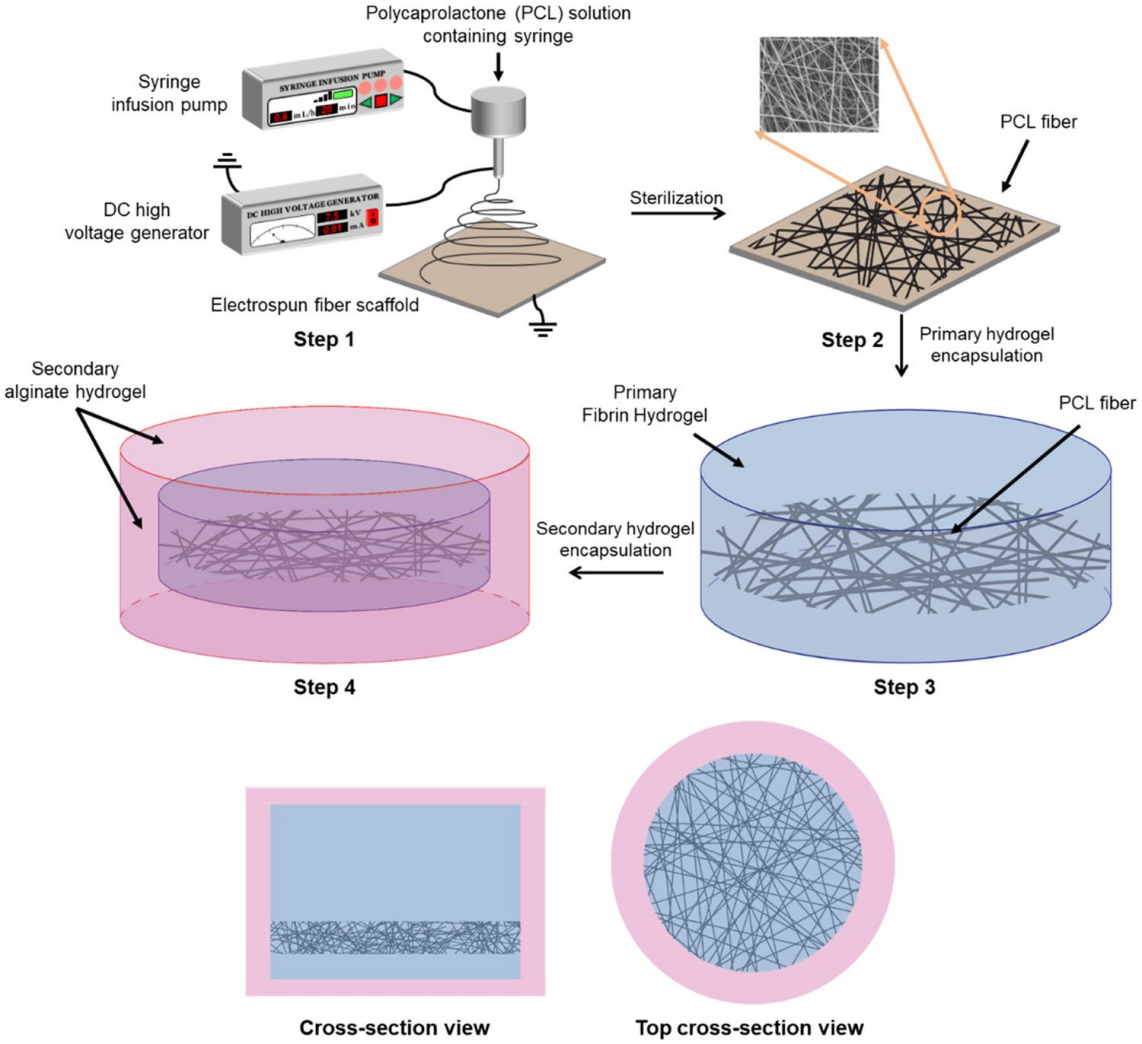
Fabrication procedure of the multiscale scaffold. The multiscale scaffold is prepared by combining electrospun fibers and two different hydrogel layers.

To fabricate the multiscale scaffolds, PCL fiber was firstly prepared by electrospinning as reported previously^7^. Briefly, 20 wt% solution of PCL (MW: 80,000; Sigma Aldrich) dissolved in 2,2,2-trifluoroethanol (TFE) was ejected using syringe pump to a 7.5 kV positive voltage supplied 18G metal needle at a constant speed (0.8 mL/h) for 20 min. The resultant PCL fibers were cut in 5 mm square shape using micro scissors and immersed in a 96-well plate containing 100 μL of fibrin precursor solution (20 mg/mL in 3 w/v % NaCl), which was crosslinked with the same amount of thrombin (2.5 U/mL in 20 mM CaCl_2_) for 15 min at 37 °C. After retrieving the PCL-fibrin hybrid scaffold using micro spoon spatula, a secondary alginate hydrogel layer was introduced to surround the fibrin hydrogel. Briefly, the retrieved PCL-fibrin hybrid scaffold was immersed in another 96 well plate containing 50 μL of 2 wt% sodium alginate hydrogel precursor solution and 15 μL of 1 wt% calcium chloride solution was added for 30 s at room temperature to crosslink alginate. Fabrication process of multiscale scaffold was described in Fig. 1.

### Characterization of multiscale scaffolds

The internal structure of multiscale scaffolds was observed using Cryo-Focused Ion Beam Scanning Electron Microscopy (cryo-FIB/SEM, Quanta 3D FEG, FEI, Netherland) with a cryo-transfer system (Alto 2500, Gatan, UK)^23^. The multiscale scaffolds were loaded to a carbon tape coated copper stub and then immersed in liquid nitrogen with a pressure of 10^–5^ mbar in a load lock chamber at approximately − 220 °C for 60 s. After a freezing process, the samples were transferred to a preparation chamber at about − 95 °C to etch the surrounding frost for a definite assay. Metal deposition with a complex of Au and Pd at a ratio of 6:4 was performed using plasma sputtering with a 3-mA current for 60 s. The frozen and metal-coated samples were transferred into a pre-exhausted microscope chamber with a pressure of 10^–5^ mbar and at a temperature of − 190 °C. The cryo-SEM images of interfaces between different regions of the multiscale scaffolds were obtained with a FIB milling process with a 5-keV energy electron beam and 47.3 pA of electron current.

To study the mechanical properties of multiscale scaffolds, the compressive modulus of fibrin hydrogel, PCL-fibrin hybrid scaffold, and multiscale scaffolds was measured using Instron 5900 (Instron corporation, USA). Each scaffold was immersed into PBS and allowed to swell for 30 min; any excessive or residual fluid was then removed before analysis. Scaffolds were then loaded between two flat plates and compressive stress and compressive strain were measured by compressing each scaffold to 80% of the existing height in the measurement. From the obtained compressive stress and strain, true stress and strain were calculated. Next, a true stress–strain curve was obtained, and the slope of the curve in the elastic region was then measured to acquire the compressive Young’s modulus of scaffolds.

### Loading of cells into the scaffold

For in vitro or in vivo studies, cells were embedded in the multiscale scaffolds compartmentally during each fabrication process. ADSCs and C2C12 cells (1.0 × 10^5^ cells each) were mixed and seeded onto the PCL fibrous matrix and cultured in incubator to attach cells on the fibrous matrix. Then, resultant PCL fiber was immersed into the fibrin precursor solution containing HUVECs (1.0 × 10^5^ cells) and crosslinked sequentially. For cell-laden multiscale scaffolds, alginate hydrogel was induced in outer layer of fibrin hydrogel. ADSC only group (2.0 × 10^5^ cells) and HUVEC only group (1.0 × 10^5^ cells) in the multiscale scaffold were used as negative controls for PCR analysis and immunostaining. These cell-laden scaffolds were cultured in different types of media depending on the cell composition: Single type of cell composition—each cell culture media, ADSC and C2C12 group—1:1 ratio of DMEM and Alpha MEM, ADSC and HUVEC group—1:1 ratio of DMEM and EGM-2, triple culture group—1:1:1 ratio of DMEM, Alpha MEM and EGM-2. The ADSCs and HUVEC were used at passage 5.

### In vitro cell migration study

To observe whether cells migrate from the inside to outside the multiscale scaffold or PCL-fibrin hybrid scaffold, only ADSCs (2.0 × 10^5^ cells) were seeded in each scaffolds and cell-laden scaffolds were cultured on 24 well plate for 2 weeks and the boundary of the scaffolds was observed using inverted microscope every week. To confirm the cell migratory capacity of the scaffolds, the region of cell migration was measured using Image J.

### Subcutaneous implantation

All procedures were approved by the WooJungBio Institutional Animal Care and Use Committee in Korea (protocol number: IACUC2018-1-12). Subcutaneous implantation was adopted to examine the cardiovascular tissue regeneration inside the scaffolds and the external immune response. Briefly, cells were cultured in the multiscale scaffolds for 1 day before subcutaneous implantation. Male C57BL/6 mice at 12 weeks were anesthetized by isoflurane inhalation (2.5% isoflurane in oxygen). An incision of about 1 cm was made on the back of mice and cell-laden scaffolds were placed subcutaneously on one or both sides based on the incision made. The skin was then closed with sutures. At 1, 2, and 3 weeks after implantation, mice were euthanized by CO_2_ inhalation, and the scaffolds and surrounding subcutaneous tissues were then obtained. The whole procedure of animal study was confirmed in accordance with the relevant Animal experiment laws and regulations of WooJungBio (LMO facility number: LML14-376).

### Quantification of CXCL-1 in blood

After euthanasia, mice were fixed and their chest was opened using operating scissors before heart arrest. After that, about 5 mL of blood was collected using a syringe and transferred into an ice bath to prohibit clotting. Serum was isolated by centrifugation at 4500 rpm at 4 °C for 10 min. The inflammatory protein CXCL-1, also known as KC (Keratinocyte-derived chemokine), was quantified using a Mouse CXCL-1/KC Quantikine ELISA kit (R&D Systems, United States).

### Quantitative reverse transcription PCR (qRT-PCR)

Total RNA was extracted from the cells in the implanted scaffolds using TRIzol (Life Technologies). One microgram of total RNA was reverse transcribed into cDNA using a Maxime RT PreMix Kit (iNtRON Biotechnology, Korea). To observe the expression of cardiac- and blood vessel-specific markers from implanted ADSCs and HUVECs, qRT-PCR was performed using the Maxime PCR PreMix kit (iNtRON Biotechnology) on an Applied Biosystems QuantStudio 5 Real-Time PCR Instrument (Thermo Fisher Scientific United States). The specific human primers used to observe gene expression are listed in Supplementary Table S1. The relative expression levels of each gene from three independent experiments conducted in triplicate was normalized to the expression of the house-keeping gene, GAPDH and was calculated using the 2^−ΔΔCT^ method.

### Immunostaining

Immunostaining of the cardiac- and blood vessel-specific markers was performed on sections of OCT compound-embedded scaffolds. Implanted cell-laden multiscale scaffolds were collected at the previously indicated time points and fixed in 10% paraformaldehyde for 10 min at room temperature, rinsed with PBS, dehydrated sequentially in 30% sucrose solutions. The resulting scaffolds were embedded in OCT compound and immersed in liquid nitrogen for rapid freezing, followed by sectioning at 9-μm thickness. Sections of scaffolds were blocked with 3% bovine serum albumin for 1 h, and then permeabilized with 0.1% Triton X-100 in PBS for 10 min at room temperature before overnight incubation at 4 °C with primary antibodies specific for CD31 (MA3100, Invitrogen), cardiac troponin T (cTnT, ab8295, Abcam), and sarcomeric alpha actinin (s. α. Actinin, ab9465, Abcam, Cambridge, UK) in 1% BSA. The sections were then washed three times with PBS, and then incubated with Alexa Fluor 568-conjugated secondary antibody (Abcam) for 1 h at room temperature in the dark. The nuclei were counterstained with 4′,6-diamidino-2-phenylindole, dihydrochloride (DAPI; Sigma Aldrich). The labeled slides were visualized using a laser scanning microscope (Carl Zeiss, Oberkochen, Germany).

To confirm the immune response of implanted scaffolds, the surrounding mice skin tissues were fixed in 10% neutral buffered formalin (Dana Korea, Incheon Metropolitan City, South Korea), washed in water, and dehydrated from low concentration to high concentration of ethanol (80% → 90% → 95% → 100% → 100%) for 1 h each. Tissues were then incubated in three increasing xylene concentrations for 1 h each. Mice colonic tissues were fixed in 10% neutral buffered formalin, paraffin-embedded, sectioned (5 μm), deparaffinized, and stained with hematoxylin and eosin (Agilent Technologies, Santa Clara, California, USA) and Masson’s trichrome (Agilent Technologies, Santa Clara, California, USA). Sections on slides were stained with hematoxylin, bluing buffer, eosin, dehydrated using alcohol, and then rinsed in xylene. Slides were stained using Masson’s trichrome kit and Dako ArtisanLink Pro (Agilent Technologies, Santa Clara, California, USA). Masson’s trichrome kit consists of Bouin’s solution, Weigert’s hematoxylin, Biebrich Scalet acid fuchsin, phos/phos acid, aniline blue, and acetic acid. The degree of inflammation in skin was evaluated according to a scoring system based on Supplementary Table S2. Slides were photographed by optical microscopy (Leica, Wetzlar, Germany) and rendered using Adobe Photoshop program and Leica software.

### Statistical analysis

Statistical analysis was conducted using the Graph Pad Prism 5 package (GraphPad Software Inc., La Jolla, CA, USA). The one-way ANOVA followed by Tukey’s post hoc test were used to compare values among groups. A p < 0.05 was considered to indicate the statistical significance.

## Results

### Characterization of the multiscale scaffolds

**Figure 2:**
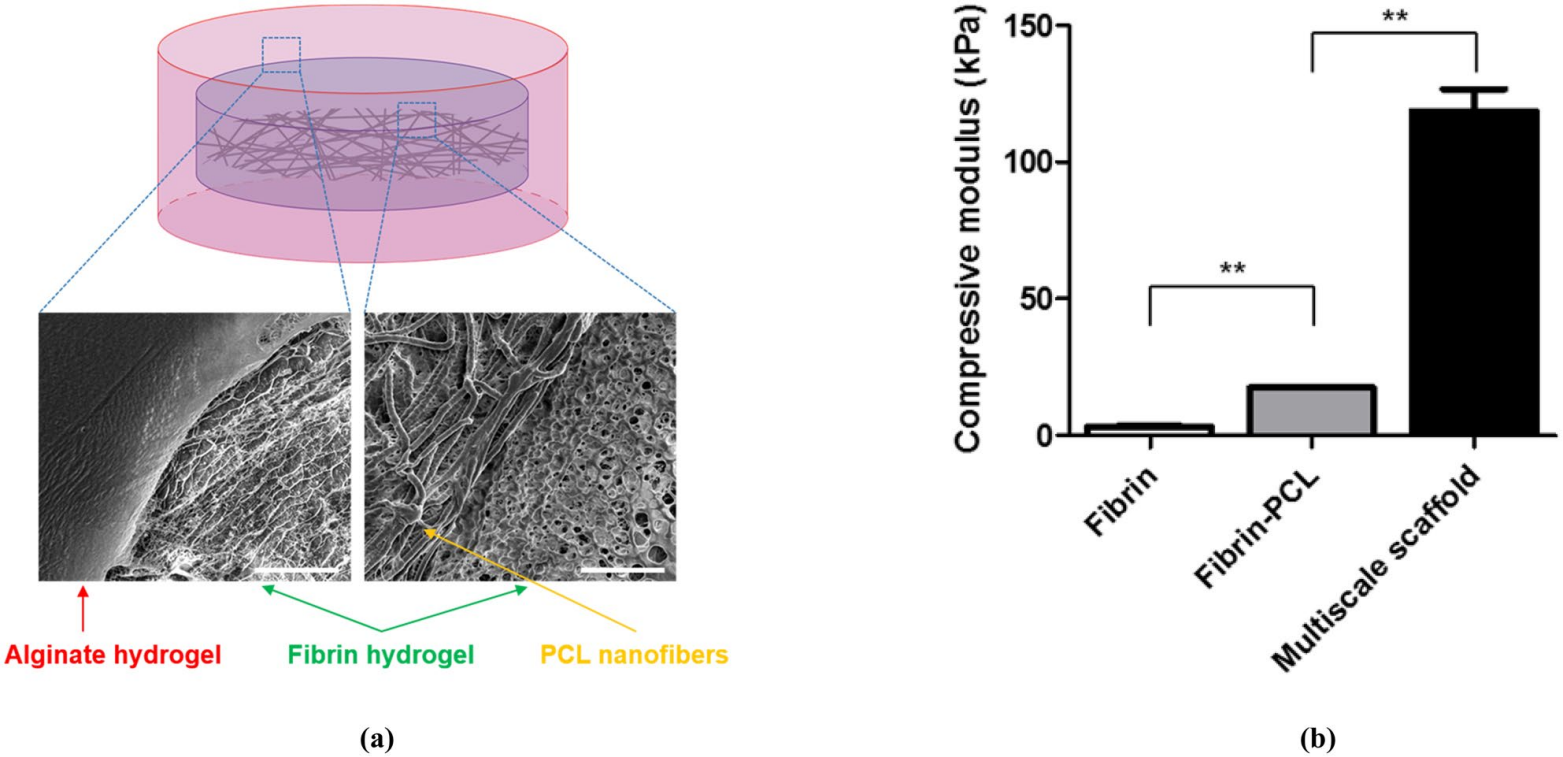
Characterization of the multiscale scaffold comprising electrospun fibers and two different hydrogel layers. Scale bar = 20 μm. (**a**) Representative cryo-SEM image revealing the compartmentalized structure of the multiscale scaffold. (**b**) Compressive modulus of scaffolds (n = 4). Data are mean ± s.d. One-way ANOVA with Tukey’s post-hoc test was used. **P < 0.01.

Before the study on cardiovascular tissue regeneration in vivo, we investigated the regenerative capacity of cells in the multiscale scaffolds in vitro. We designed multiscale scaffolds, where three different cell types (ADSCs, HUVECs, and C2C12) were compartmentalized in different regions. We expected that the resultant triple culture system would promote stem cell differentiation into cardiac lineages along with peripheral blood vessel formation to construct in vitro cardiovascular tissue as described in GA. To prepare multiscale scaffolds for the cell study, we combined a conventional electrospinning process and hydrogel fabrication method (Fig. 1). The designed final multiscale scaffold comprises fibrin hydrogel containing electrospun fiber at the bottom with surrounding alginate hydrogel. Cryo-SEM images of the multiscale scaffolds revealed that the PCL fibers are located in the fibrin hydrogel and that the secondary alginate hydrogel surrounds fibrin hydrogel (Fig. 2a). PCL fibers with a diameter of about 800 nm could be obtained from the electrospinning process, which allows attachment and proliferation of ADSCs and C2C12. In case of two layers of hydrogels, the inner fibrin hydrogel was observed as a porous structure and the outer alginate hydrogel had a smooth surface. With the addition of electrospun PCL fibers and alginate hydrogel to fibrin hydrogel in each fabrication step, increased compressive Young’s modulus of the scaffold was observed. The compressive Young’s modulus of fibrin hydrogel alone increased from 3.10 ± 0.995 kPa to 17.17 ± 0.326 kPa when the PCL fiber was encapsulated. With the surrounding alginate hydrogel, the final compressive modulus of the multiscale scaffold was increased to 118.660 ± 15.973 kPa (Fig. 2b). These findings suggest that the multiscale scaffold can be mechanically stable enough to support cell attachment and proliferation during culture periods with compartmentalization of different types of cells in the fibrin hydrogel and PCL fiber, respectively.

### Cardiac phenotypic expression of cells in the multiscale scaffold

**Figure 3:**
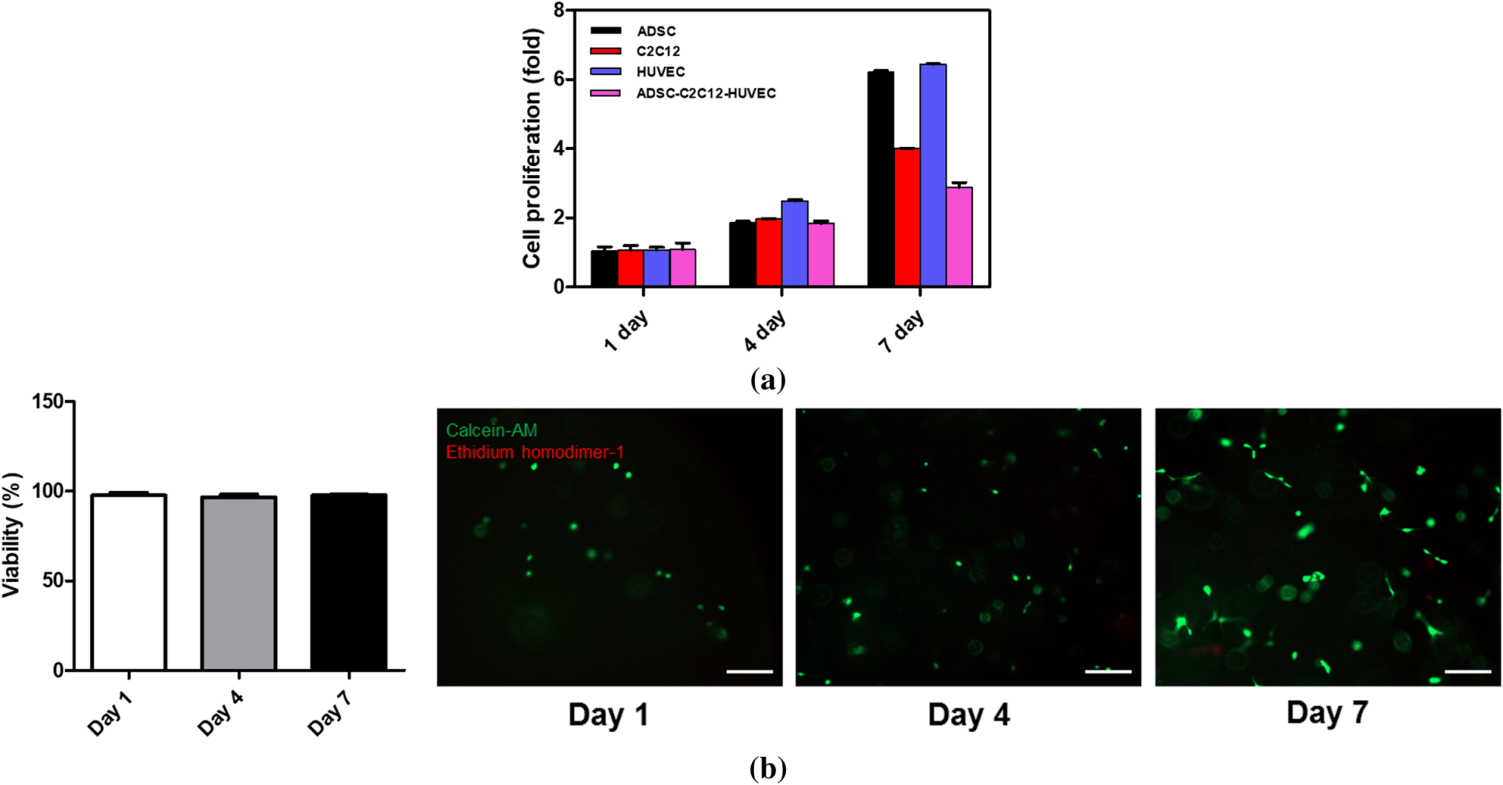
Cytocompatibility of the multiscale scaffold. (**a**) CCk-8 analysis using different cell types cultured in multiscale scaffolds. (**b**) Viability of HUVECs for 7 days (left) and representative LIVE/DEAD images of HUVECs in the fibrin hydrogel (right).

Next, we investigated cell behavior in the multiscale scaffold and PCL-fibrin hybrid scaffold. For cell study, ADSCs and C2C12 were seeded on a PCL fibrous mesh positioned in the bottom of the fibrin hydrogel, whereas HUVECs were seeded in the fibrin hydrogel region. CCK-8 results showed that all mono-cultured and triple-cultured cells proliferated continuously without any significant cell death up to 7 days (Fig. 3a). The cytotoxicity of fibrin hydrogel was confirmed through a LIVE/DEAD assay of the seeded HUVECs, where viability was calculated by the number of living cells/total number of cells. Fluorescence images revealed that most cells were alive and able to proliferate within the fibrin hydrogel, indicating a low cytotoxicity of the fibrin gel and gelation process (Fig. 3b).

**Figure 4:**
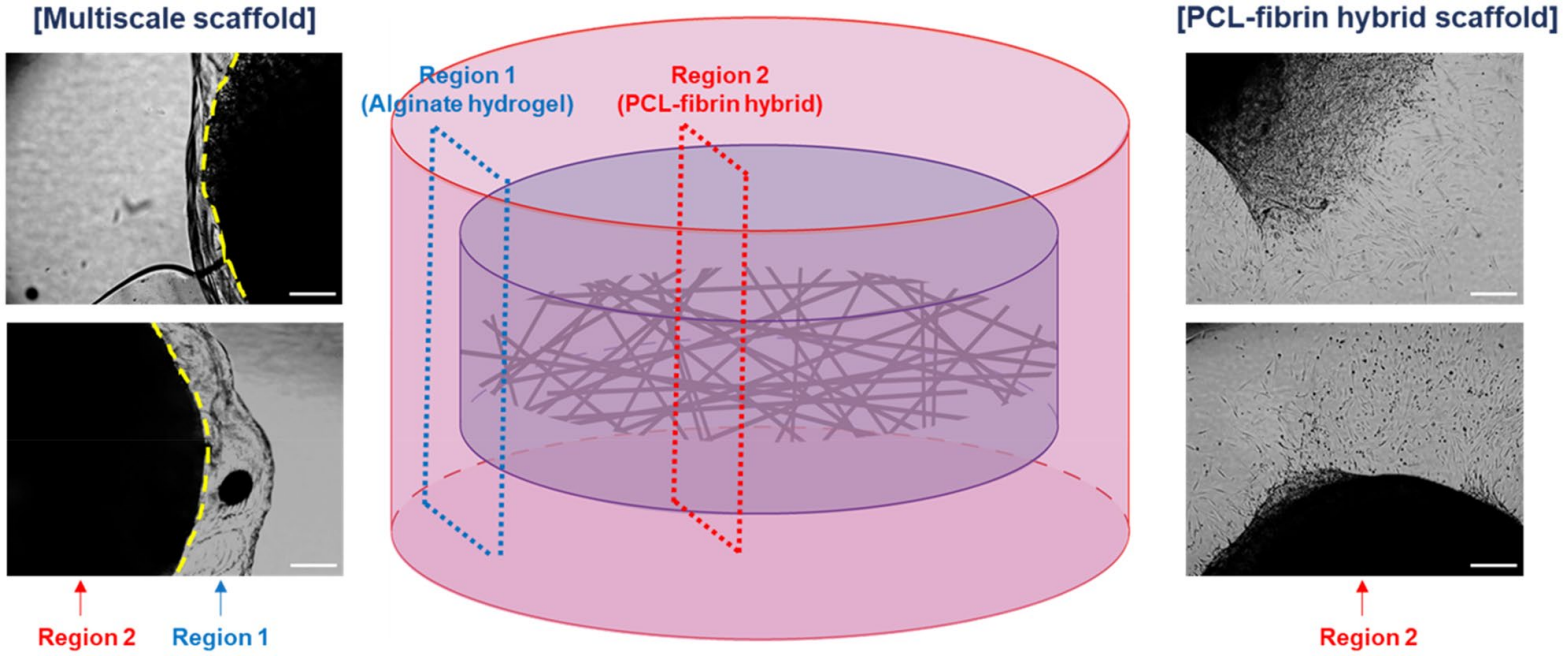
Effect of secondary alginate hydrogel layers on the migration of cells encapsulated in the PCL-fibrin hybrid scaffold. Cells in the multiscale scaffold and PCL-fibrin hybrid scaffold were observed at 2 weeks after culture using an inverted microscope. (left) Microscope images for boundary between alginate hydrogel layer and fibrin hydrogel in the multiscale scaffold. (middle) A schematic illustration of multiscale scaffold and indication of observation site. (right) Microscope images of HUVECs migration from PCL-fibrin hybrid scaffold to outside. Scale bar = 200 μm.

Given that the designed multiscale scaffolds could support construction of cardiovascular tissue, we compared ADSCs seeded in the multiscale scaffold and the PCL-fibrin hybrid scaffold without alginate hydrogel to observe cell migration from inside the scaffold to outside (Fig. 4). The cells in the PCL-fibrin hybrid scaffold escaped and migrated to the outside. However, with the addition of secondary alginate hydrogel, cells migrated only inside the fibrin hydrogel, but no migration to alginate hydrogel or outside the scaffold was observed, as confirmed from the optical microscope images.

**Figure 5:**
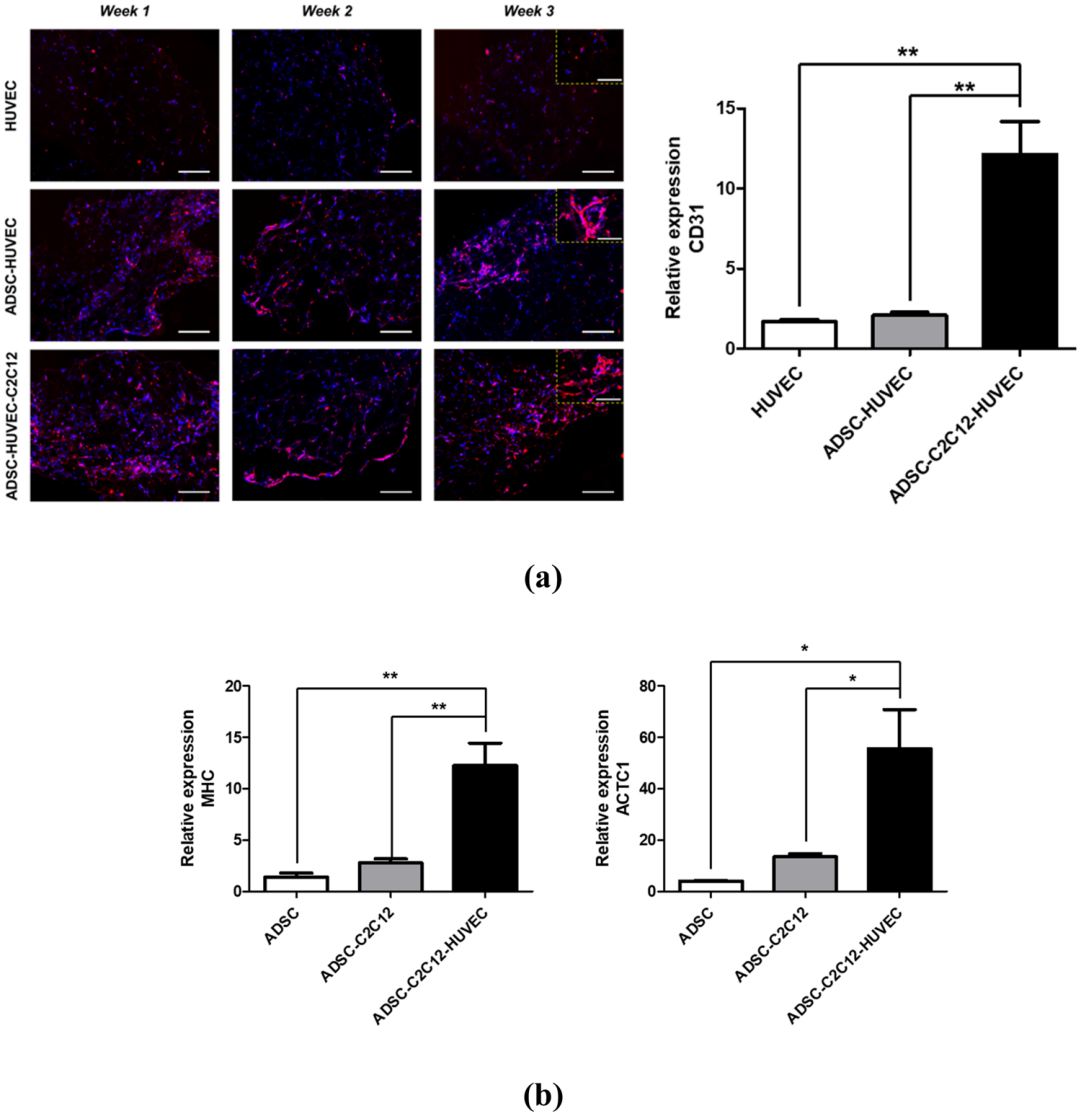
In vitro cardiovascular tissue formation in the multiscale scaffold. (**a**) (left) Immunofluorescence images for CD31 in the fibrin hydrogel region of the multiscale scaffold at each time point. Yellow dashed rectangle insets show × 40 magnified images of each group. Scale bars = 200 μm and 100 μm in low magnification and high magnification images, respectively. (right) Quantitative analysis of CD31 expression by qRT-PCR at 3-week culture. (**b**) Quantitative expression of cardiac markers (MHC, ACTC1) from ADSCs in the multiscale scaffold by qRT-PCR at 3-week culture. The expression levels to relative to the housekeeping gene (GAPDH) were calculated for analysis of qRT-PCR results (n = 3). Data are mean ± s.d. One-way ANOVA with Tukey’s post-hoc test was used. ***P < 0.001, **P < 0.01.

We further investigated blood vessel formation from HUVECs and cardiomyogenic phenotype expression of ADSCs in the multiscale scaffold before in vivo implantation. From the immunofluorescence staining images of CD31, some CD31^+^ cells were observed in all groups after 1 week, but luminal structures became visible from HUVECs after 2 weeks after seeding only in HUVECs and ADSCs co-cultured group and triple-culture group. During the culture periods, in the co-cultured scaffold of HUVECs and ADSCs, more CD31^+^ cells were found compared to the mono-cultured scaffold, and the most CD31^+^ cells were observed in the triple culture condition. However, in qRT-PCR results, gene expression of CD31 in triple-culture group was significantly higher than other groups, and statistical meaning between HUVECs monocultured group and co-cultured group was not found at 3-week culture (Fig. 5a). To confirm the cardiomyogenic fate of ADSCs in the multiscale scaffold, qRT-PCR was performed for cardiac specific markers (MHC, ACTC1). By co-culturing ADSCs with C2C12, the expression of two human cardiac markers slightly increased, compared to that of the monocultured ADSCs and significantly higher expression was observed in triple culture condition. (Fig. 5b). In summary, tripled-cultured ADSCs were able to induce blood vessel formation of HUVECs as demonstrated in a previous study^24^ and were expected to regenerate cardiac tissue through cardiomyogenic lineage markers expression.

### Cardiovascular tissue regeneration in the scaffold after subcutaneous implantation

**Figure 6:**
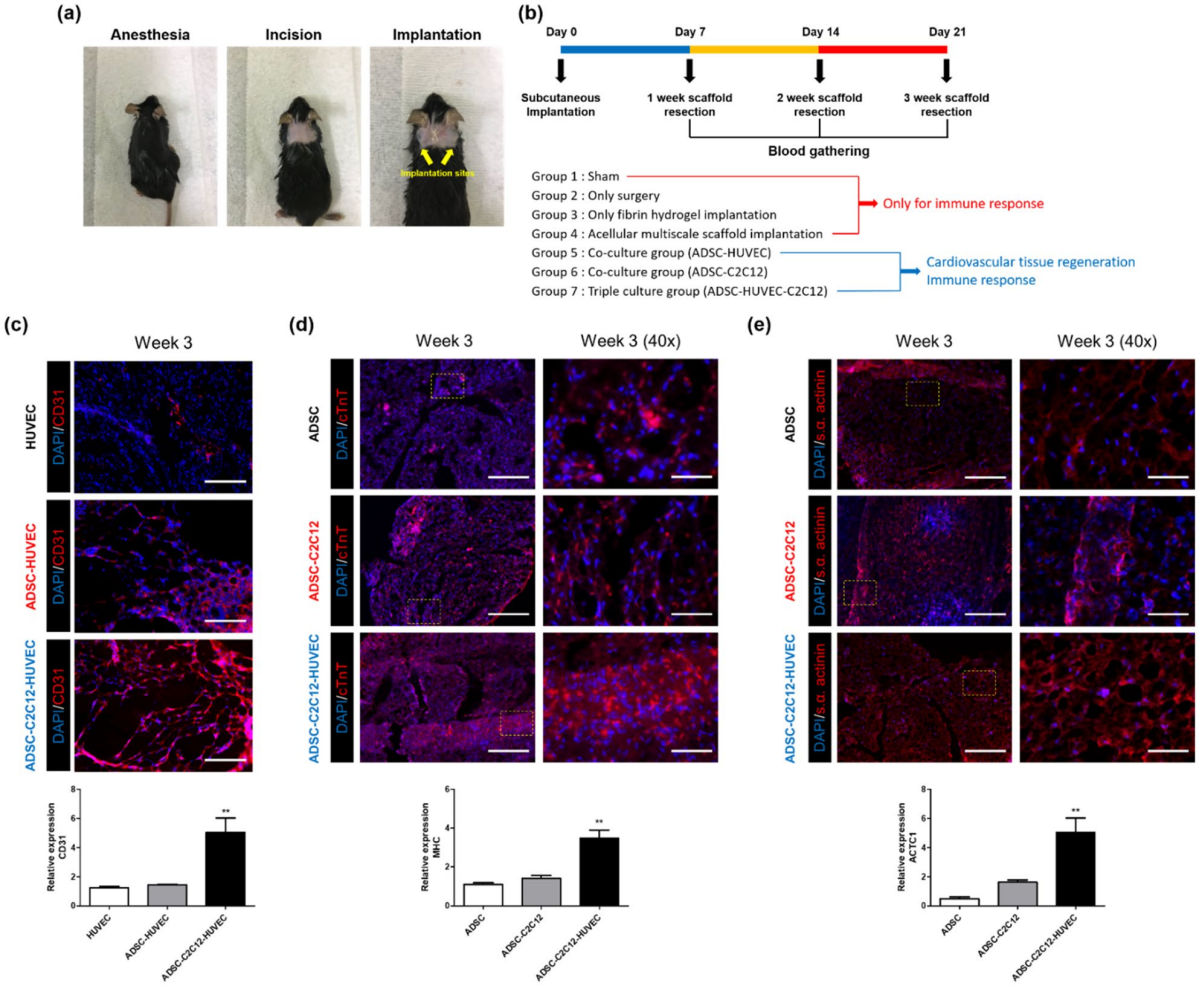
Cardiovascular tissue regeneration capacity of cell-laden multiscale scaffolds after subcutaneous implantation. (**a**) Photos showing the experimental procedure for in vivo studies. (**b**) Description of experimental design for in vivo studies. Immunofluorescence images and qRT-PCR results for (**c**) CD31, (**d**) cTNT, and (**e**) s.α. actinin. Scale bar = 200 μm and 50 μm for low magnification images and high magnification images, respectively. Data are mean ± s.d. One-way ANOVA with Tukey’s post-hoc test was used, compare to ADSC and HUVEC monocultured group, respectively. ***P < 0.001, **P < 0.01.

To evaluate the effect of the multiscale scaffold toward forming an implantable cardiovascular tissue regeneration system, triply seeded multiscale scaffolds were placed subcutaneously in C57BL/6 mice and evaluated weekly until 3 weeks after implantation. The implanted scaffolds were placed one on each side of the vertebra (Fig. 6a). To compare the regeneration effect of the triple culture system, we also implanted mono-cultured scaffolds (HUVECs only and ADSCs only) and co-cultured scaffolds (ADSCs-HUVECs and ADSCs-C2C12). Furthermore, we compared the acellular multiscale scaffold and bare fibrin hydrogel to identify the effect of secondary alginate hydrogel on maintaining the whole construct after implantation. Experimental design for in vivo studies was described in Fig. 6b. Absence of alginate hydrogel layer was found to result in melting and disappearance of the fibrin hydrogel (data not shown).

**Figure 7:**
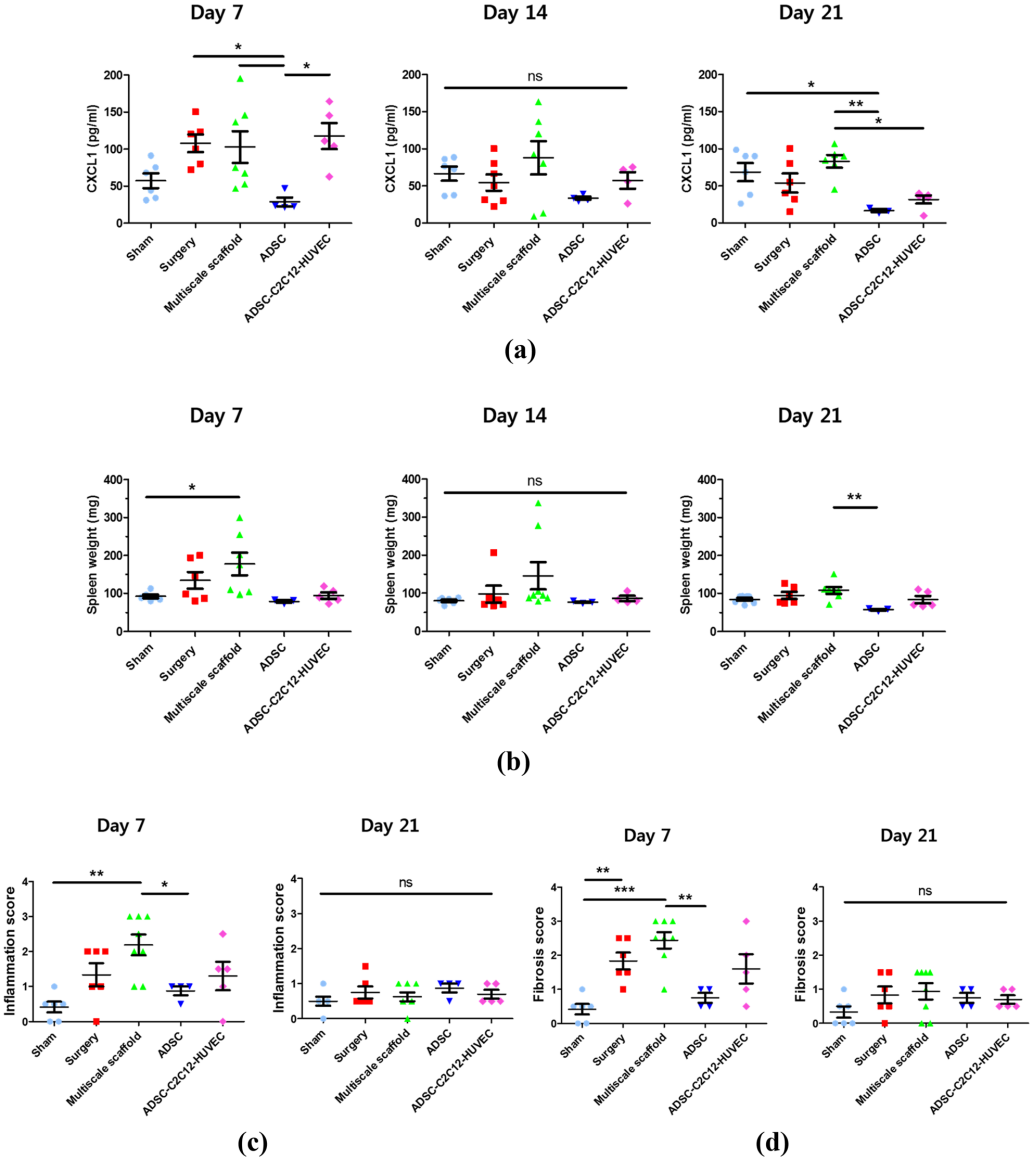
Immune response after subcutaneous implantation. (**a**) Photos showing experimental procedure for blood collection from the heart. (**b**) Concentrations of CXCL-1 in blood at day 7, 14, and 21 after implantation. (**c**) Spleen weights of C57BL/6 mice implanted with multiscale scaffolds. (**d**) Inflammation score. (**e**) Fibrosis score Data are mean ± s.d. One-way ANOVA with Tukey’s post-hoc test was used. ***P < 0.001, **P < 0.01, *P < 0.05, *ns* no statistical significance. Individual results are presented and the mean for each group is indicated by a straight line in all graphs.

At 3 weeks, the multiscale scaffolds containing triple-cultured cells showed the highest expression of blood vessel-specific markers and cardiac markers in the peripheral fibrin hydrogel region and core fiber mesh of the scaffold, respectively as shown in Figs. 6c,d, and 7e (data obtained after 1 and 2 weeks are shown in Supplementary Fig. S1). Specifically, significant blood vessels and capillary-like structures appeared in the fibrin hydrogel region, only in the triple-cultured group, indicated by a substantial increase in the assembled structure of CD31 positive cells (fluorescence images in Fig. 6c). A similar result was observed for the gene expression of CD31 as in the in vitro studies shown in Fig. 6a. Fluorescence immunohistochemistry of cardiac specific markers (cTnT, s.α. actinin) in implanted scaffolds showed remarkable regeneration of cardiac tissue in the implanted triple cultured groups after 3 weeks of implantation (fluorescence images in Fig. 6d,e). Based on these results, we focused on investigating the resultant expression of cardiac phenotype from transplanted ADSCs using qRT-PCR with human specific primers (Fig. 6d,e). Expression of both cardiac markers (MHC, ACTC1) was enhanced in ADSCs-C2C12 co-cultured and triple-cultured groups up to 3 weeks after implantation, indicate a significant cardiomyogenic phenotype of ADSCs. In contrast, the transplanted mono-cultured ADSCs encapsulating multiscale scaffolds had no remarkable myocardial regeneration. These findings, therefore, indicate that the triple culture system in multiscale scaffolds is effective for cardiovascular tissue regeneration and may be applicable to the regeneration of cardiovascular tissue.

### Immune response of cell-laden multiscale scaffolds during cardiovascular tissue regeneration

We also investigated the immune response of cell-laden implanted multiscale scaffolds to explore their immune-suppressive property by analyzing the level of CXCL-1 in blood and staining immune cells at each time points. After euthanasia, we fixed the mouse and the tissue of the vicinity of the heart was opened; then, about 5 mL of blood was sampled using a syringe before the heart stopped and serum was separated to quantify the amount of the CXCL-1. The quantified amount of CXCL-1 tended to decrease over time up to 3 weeks in all groups, especially in the ADSC monoculture group, which showed the lowest protein level compared to other groups. In the triple culture group, the highest amount of CXCL-1 was shown at 1 week, but decreased gradually, and at 3 weeks after implantation, a similar level to the ADSC monoculture group was observed. However, the acellular multiscale scaffold implanted group showed the highest CXCL-1 level after 3 weeks (Fig. 7a). Different to the CXCL-1 measurement results, the triple culture group showed similar spleen weight to the ADSC monoculture group for 3 weeks. The acellular multiscale scaffold group showed the highest spleen weight after 1-week of implantation, which was significantly reduced to a level similar to other groups at the 3-week measurement (Fig. 7b).

Histological sections were taken from the peripheral skin of the implanted multiscale scaffold in a transverse section. We scored the degree of immune response and fibrosis of the implanted scaffolds under the criteria of representative images (Supplementary Table S2 and Fig. S2). Histological assessment demonstrates that the ADSC monoculture group showed a relatively low immune score and fibrosis score compared to other groups, conversely, with the highest scoring level of the acellular multiscale scaffold at 1-week observation after implantation (Fig. 7c,d). The triple culture group showed a slightly higher level of immune response compared to the ADSC monoculture group after 1 week of implantation; however, a similar observation was obtained thereafter. At the 3 week’s observation after implantation, suppressed immune response was observed in the acellular multiscale scaffold to the level of all groups, which represents a moderate inflammatory response (Fig. 7c,d and Supplementary Figs. S3–S5).

## Discussion

We fabricated multiscale, hybrid, and implantable scaffolds that are mechanically stable and capable of reducing immune responses after subcutaneous implantation. The compartmentalized triple-culture system was applied to induce stem cells into cardiac phenotype and peripheral blood vessel formation, which was confirmed to induce new cardiovascular tissue inside the scaffolds (GA). The multiscale scaffolds were fabricated by combining a conventional electrospinning process and sequential fabrication of two hydrogel layers with different properties (Fig. 1): a macroporous and soft primary fibrin hydrogel region containing the PCL fiber at the bottom, and a secondary alginate hydrogel protecting the PCL-fibrin hybrid scaffold. We hypothesized that this structural support could enhance cardiovascular tissue formation in the multiscale scaffold mimicking the natural tissue environment. Notably, our system showed cardiac characteristics of stem cells and peripheral blood vessel formation, which imply a future engineered tissue transplantation system for clinical applications. A previous study showed that pre-vascularization of engineered skeletal muscle tissue improved in vivo vascularization and cell survival, which could induce the regeneration capacity in engineered tissues^25^. Consistent with this notion, our triple culture system in the multiscale scaffold including stem cells, myoblasts, and endothelial cells is unique and we investigated the feasibility of new cardiovascular tissue regeneration in the scaffold both in vitro and in vivo.

Our data show that the resulting multiscale scaffolds have suitable mechanical properties as natural myocardium to support cell transplantation until mature cardiovascular tissue formation due to the secondary alginate hydrogel and compartmentalized structure dividing the cell culture region (Fig. 2)^26^. From the perspective of mimicking actual cardiovascular tissue, this system was designed to achieve the formation of myocardial tissues and peripheral blood vessels without randomly culturing three kinds of cells inside the scaffolds. This cell culturing system allows cardiomyogenic phenotype of stem cells through direct interaction with cardiomyocytes as well as the formation of vasculature by paracrine interaction between ADSCs and HUVECs.

Since stem cell migration from the scaffold to the defect site before complete differentiation into organotypic cells is one of the problems for successful tissue regeneration, we observed the migration of ADSCs within the multiscale scaffold (Fig. 4). Introduction of the secondary alginate hydrogel prohibited the cell migration to outside because alginate hydrogels have a small pore size and non-adhesive property to cells due to the absence of binding ligands^27–29^ which might also affect immune cell adhesion and reduce host immune response after implantation. Indeed, in many experiments, cells cultured in biomaterial-based scaffolds often migrate to the outside before producing complete tissue-specific ECM after implantation to the defect site. Therefore, secondary alginate hydrogels are important to maintain the structure of multiscale scaffolds during cardiovascular tissue formation inside the scaffold.

Moreover, without any addition of growth factors or cytokines, stem cells can exhibit cardiomyogenic behavior in the scaffolds (Figs. 5b, 6d,e). Crosstalk between xenogenic or allogenic cardiomyocytes and stem cells can induce cardimyogenic differentiation but not all cardiomyocyte-like properties^30–32^. Likewise, both our in vitro and in vivo results reveal that stem cells underwent gradual transition towards the cardiac phenotype, with significantly increased gene expression of human MHC and ACTC1. In particular, the transplanted triple cultured multiscale scaffold showed significant expression levels of cardiac markers (Fig. 6e). Crosstalk through direct contact and soluble factors between co-cultured ADSCs and C2C12 induced cardiac muscle features in the scaffold. During the implantation periods, stem cell may have induced myogenesis of C2C12 via Notch-1 signaling^33^, and we confirmed ADSCs also expressed more cardiac specific gene expression when co-cultured with myoblasts. Furthermore, presence of stem cells in the scaffolds enhanced the vessel-like structures of HUVECs, which could promote cell survival and tissue regeneration by secreting angiogenic factors (e.g. VEGF, IL-6, and CXCL12) (Figs. 5a, 6c)^34,35^. The resulting vascularized engineered tissue could massively increase the number of transplantable cells with high survival rates after transplantation, and be an important factor in constructing the whole organ, which might resolve the defect site morbidity.

We firstly hypothesized that the immune response of multiscale scaffolds could be reduced compared to the bare fibrin hydrogel because of the non-cell adhesive property of alginate. Unexpectedly, however, the acellular multiscale scaffold had a higher level of immune response compared other groups (Fig. 7 and Supplementary Figs. S3–S5). These results were due to the fact that alginate promotes activation of innate immune responses through the activation of macrophage-like cells because of the M- and G-blocks in its structure as reported in previous studies^36^. Conversely, fibrin hydrogel was reported to prevent inflammatory cytokine secretion from macrophages whereas fibrinogen induced inflammatory cytokine secretion^37^. These findings of previous studies support our results of the immune response, which showed the highest level in acellular multiscale scaffolds after implantation. Although innate immune response was activated without any cell transplantation, the delivered stem cells reduced the host immune response by secreting anti-inflammatory cytokines such as TGF-β and IL-10^38^. Therefore, the transplanted ADSCs could reduce the immune response of acellular multiscale scaffold after implantation, whereas co-culture groups and the triple cultured group showed slightly higher immune responses compared to the ADSC monoculture group owing to less secretion of immunomodulatory cytokines. This is because stem cell lost immunosuppressive properties, whereas cardiac phenotype emerged in the early implantation stage. Furthermore, further study to prove accurate observation about stem cell differentiation into cardiac cell lines is needed.

## Conclusions

Here, we developed biocompatible multiscale scaffolds consisting of PCL fiber-entrapping fibrin hydrogel surrounded by alginate hydrogels. The resultant multiscale scaffolds were used for a stem cell-based triple culture system for potential application as a versatile in vitro engineered vascularized tissue transplantation platform. In vitro studies revealed that ADSCs in the resultant scaffolds promoted new blood vessel formation as well as, improved cardiac phenotype which was also confirmed from in vivo studies after subcutaneous implantation of scaffolds for up to 3 weeks. Furthermore, the triple culture system resulted in a low innate immune response. Presence of the alginate layer prevented scaffolds from disappearing by degradation and prevented encapsulated cells from migrating outside the scaffolds. We are confident that these results can provide guidelines for the construction of 3D vascularized tissues that can regulate stem cell differentiation into various organotypic cells by co-culturing with the target organ cells. However, lowering the immune-stimulating property of alginate and controlling the degradation of scaffolds are remaining challenges for successful interaction between the engineered and host tissues.

## Supplementary information

Supplementary Information.

## Data Availability

The data generated and analyzed during current study are available from the corresponding author on reasonable request.
