# Supplementary material for: Cardiovascular tissue regeneration system based on multiscale scaffolds comprising double-layered hydrogels and fibers
Source: Sci Rep. 2020 Nov 23;10:20321. doi: 10.1038/s41598-020-77187-8 (PMC7683622; doi:10.1038/s41598-020-77187-8)
Supplement: Supplementary file 1 — Supplementary Information. [file 41598_2020_77187_MOESM1_ESM.docx]

**Supplementary Information**

**Cardiovascular tissue regeneration system based on multiscale scaffolds comprising double-layered hydrogels and fibers**

Yun-Min Kook^1^, Soonjae Hwang^4^, Hyerim Kim^2^, Ki-Jong Rhee^4^, Kangwon Lee^2,3*^, Won-Gun Koh^1*^

^1^ Department of Chemical and Biomolecular Engineering, Yonsei University, 50 Yonsei-ro, Seodaemun-gu, Seoul 120-749, Republic of Korea

^2^ Program in Nanoscience and Technology, Graduate School of Convergence Science and Technology, Seoul National University, Seoul, Republic of Korea

^3^ Advanced Institutes of Convergence Technology, Gyeonggi-do, Republic of Korea

^4^ Department of Biomedical Laboratory Science, College of Health Sciences, Yonsei University at Wonju, Wonju, Gangwon-do 220-710, Republic of Korea

**Supplementary figure legends**

**Supplementary table S1.** Primer sequences used in this study for qRT-PCR.

**Supplementary table S2.** Scoring of histopathological evaluation on mouse skin.

**Supplementary figure S1.** 1-week and 2-week observations of cardiovascular tissue formation in the multiscale scaffold after implantation via immunostaining of (a) CD31, (b) cTnT, and (c) s.α. actinin.

**Supplementary figure S2.** Representative images based on inflammation and fibrosis score.

**Supplementary figure S3.** (a) H&E staining and (b) masson’s trichrome staining of cryosectioned surrounding mouse tissue at 1 week. Scale bar = 200 μm. Experiments were performed with at least five mice per groups.

**Supplementary figure S4.** (a) H&E staining and (b) masson’s trichrome staining of cryosectioned surrounding mouse tissue at 2 weeks. Scale bar = 200 μm.

**Supplementary figure S5.** (a) H&E staining and (b) masson’s trichrome staining of cryosectioned surrounding mouse tissue at 3 weeks. Scale bar = 200 μm.

**Supplementary figures & tables**

**Supplementary table S1.**

**
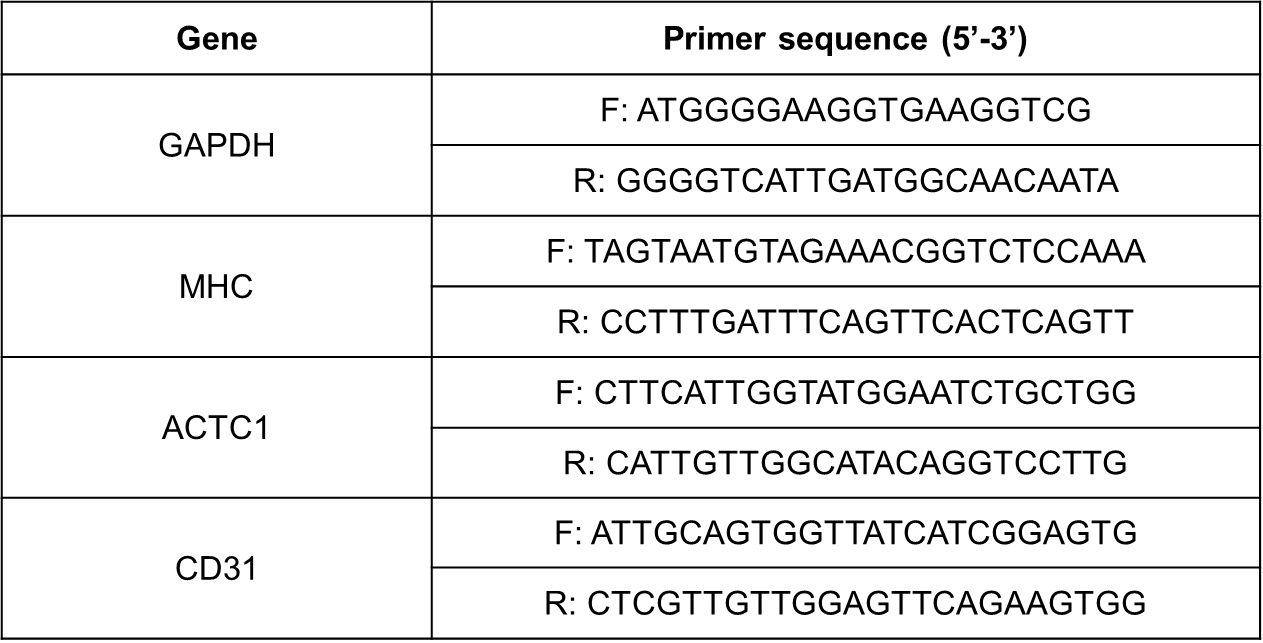
**

**Supplementary table S2.**

**
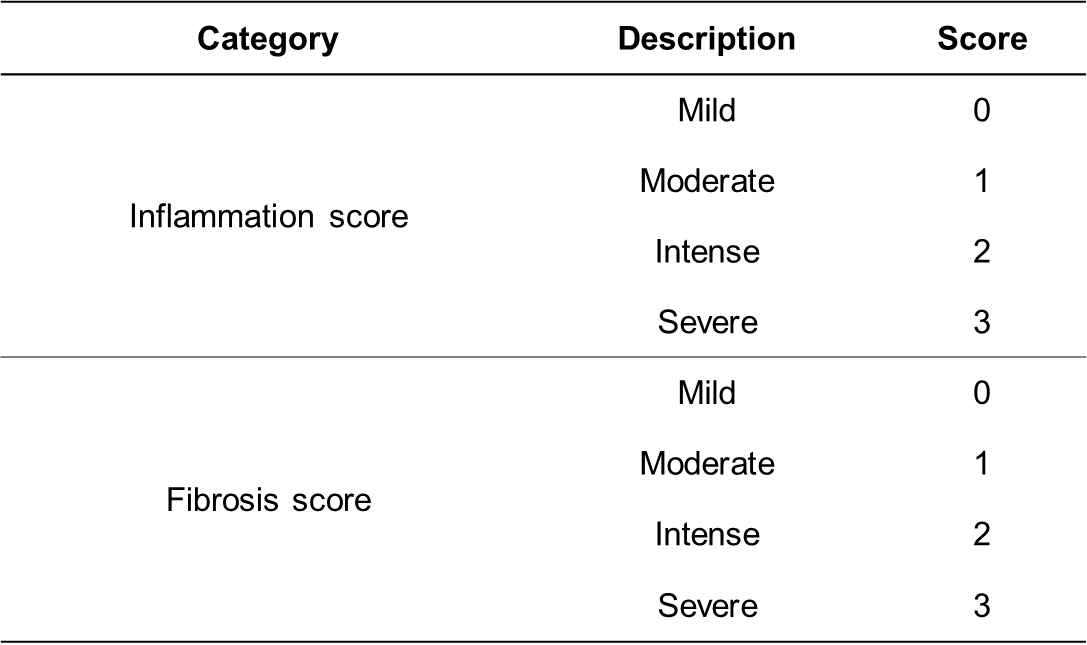
**

**Supplementary figure S1.**

**
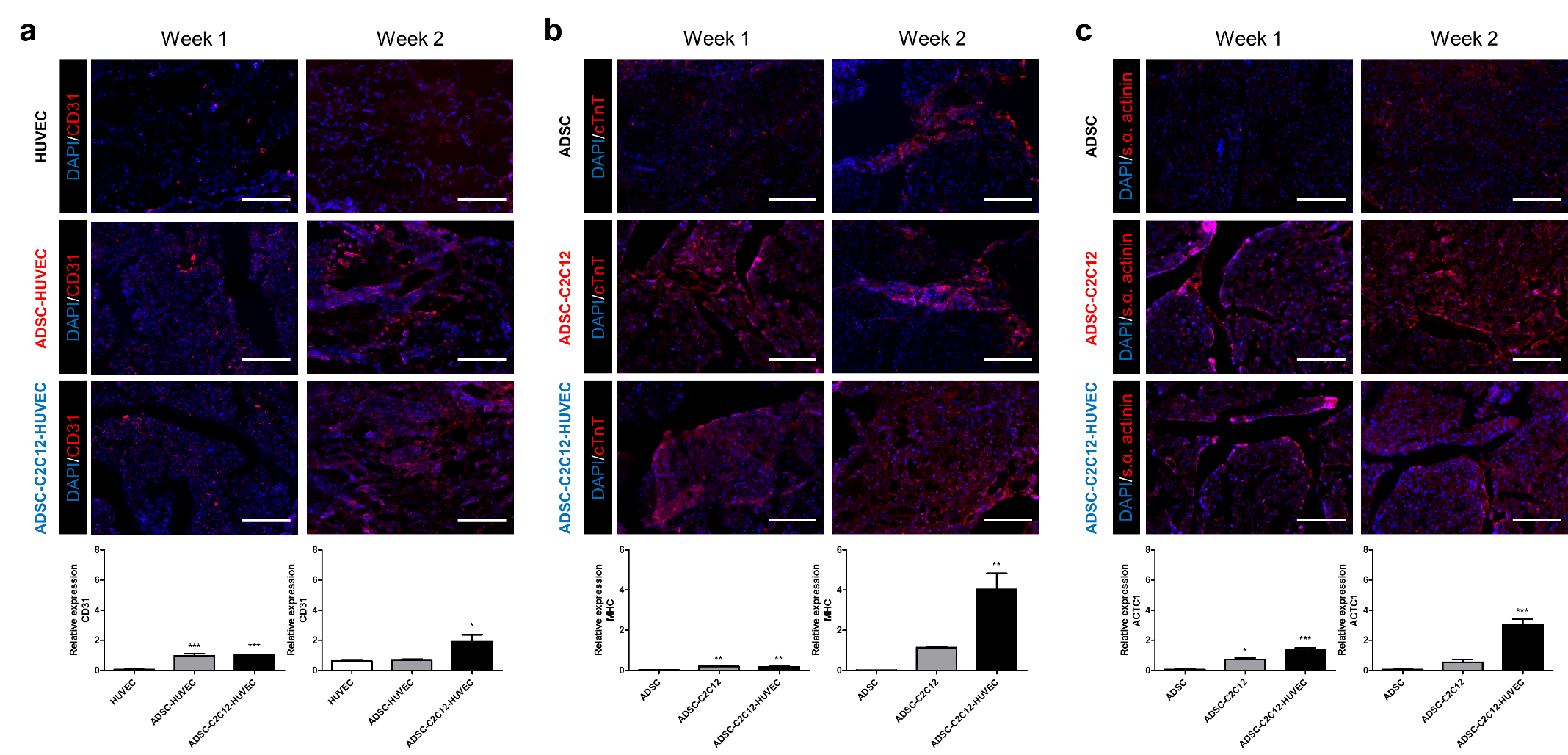
**

**Supplementary figure S2.**

**
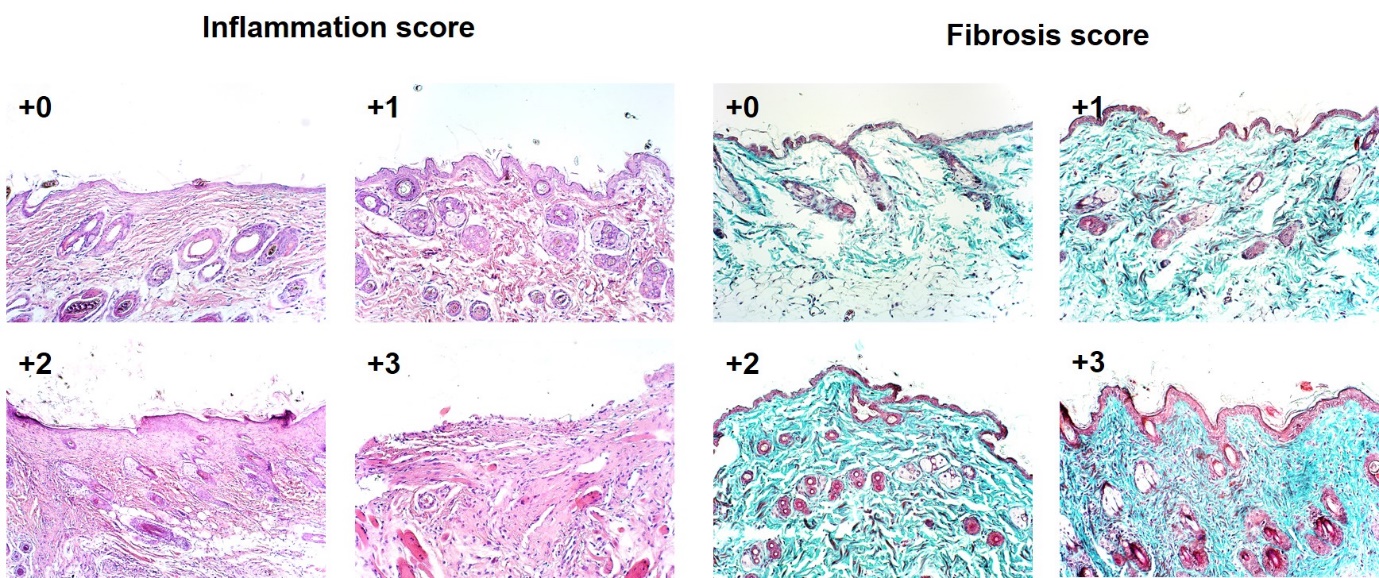
**

**Supplementary figure S3.**

**
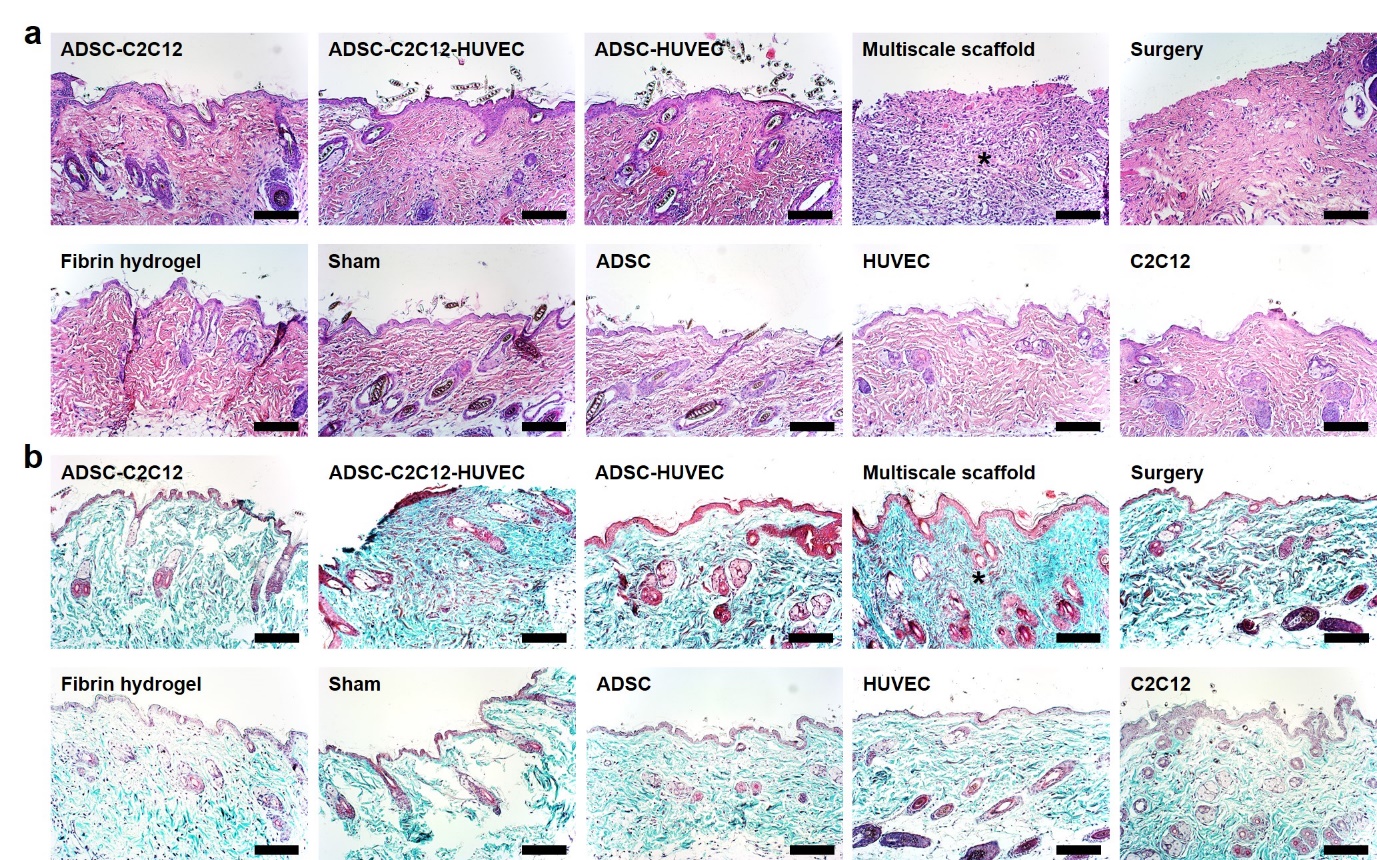
**

**Supplementary figure S4.**

**
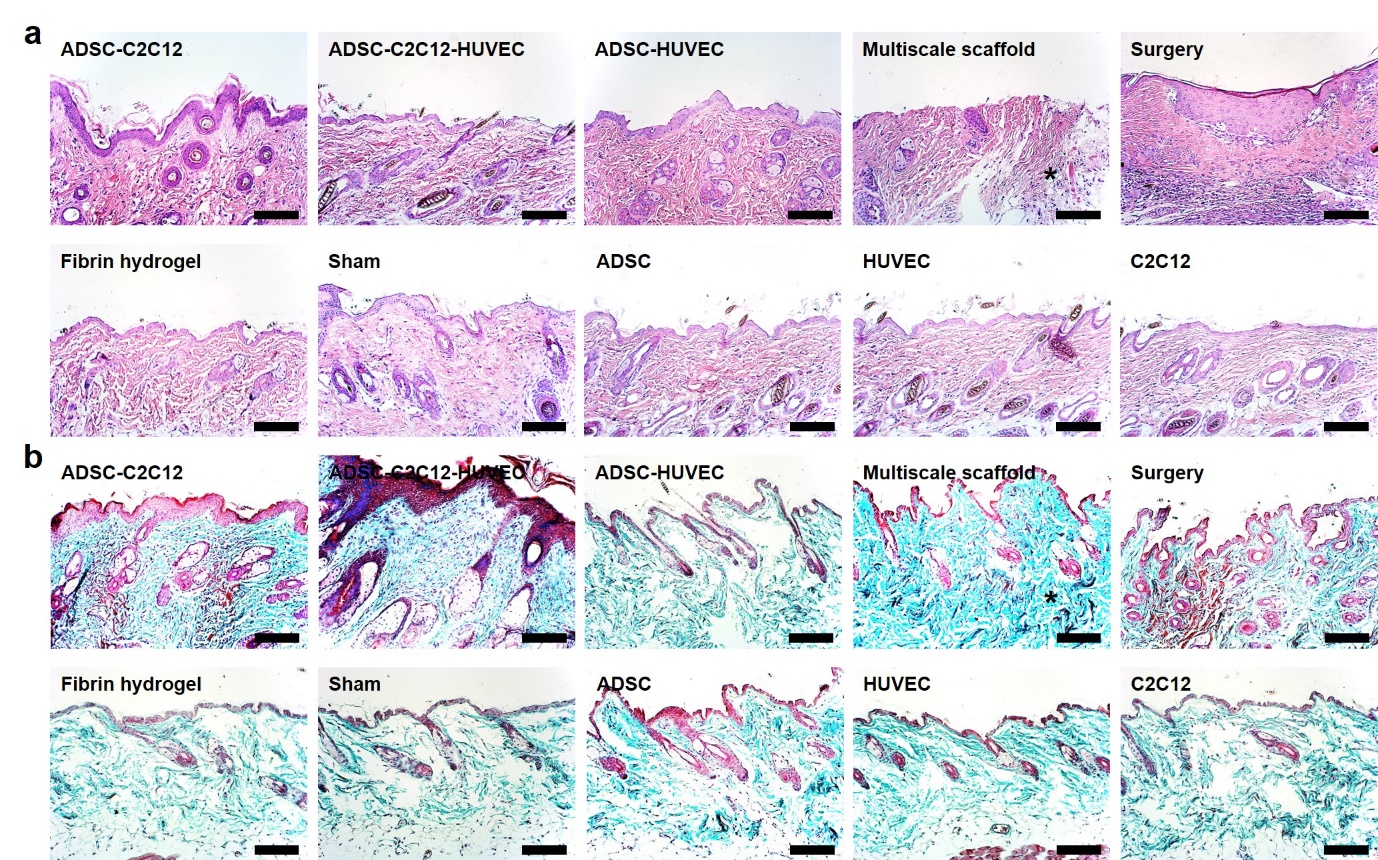
**

**Supplementary figure S5.**

**
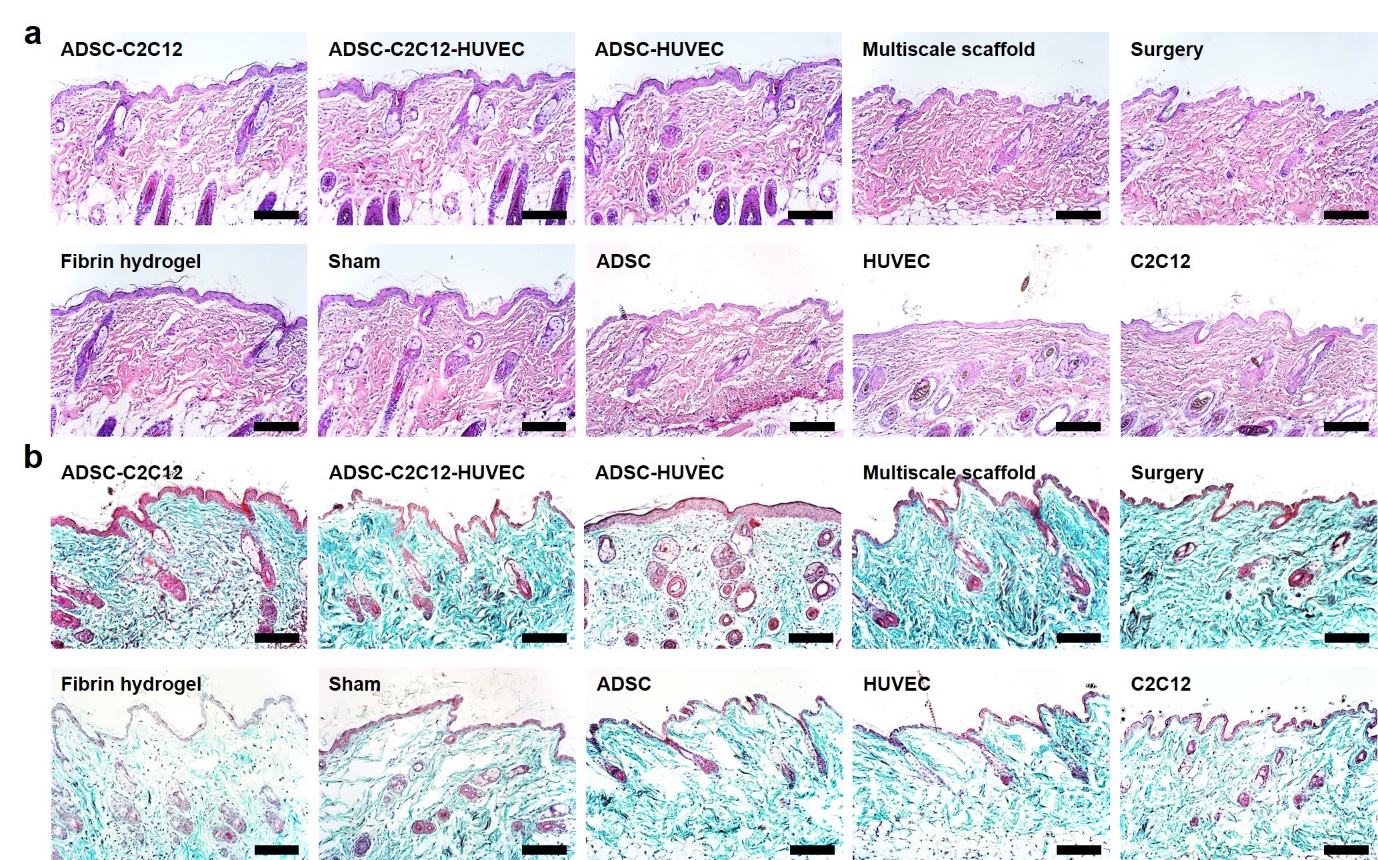
**
